# *Bax* Contributes to Retinal Ganglion Cell Dendritic Degeneration During Glaucoma

**DOI:** 10.1007/s12035-021-02675-5

**Published:** 2022-01-05

**Authors:** Michael L. Risner, Silvia Pasini, Nolan R. McGrady, David J. Calkins

**Affiliations:** Vanderbilt Eye Institute, Department of Ophthalmology and Visual Sciences, Vanderbilt University Medical Center, AA7103 MCN/VUIIS, 1161 21st Ave. S., Nashville, TN 37232 USA

**Keywords:** Bcl2-associated X protein, *Bax*^-/-^, Neuroprotection, Neurodegeneration, Glaucoma, Retinal ganglion cells, Dendritic morphology, Axonopathy

## Abstract

The BCL-2 (B-cell lymphoma-2) family of proteins contributes to mitochondrial-based apoptosis in models of neurodegeneration, including glaucomatous optic neuropathy (glaucoma), which degrades the retinal ganglion cell (RGC) axonal projection to the visual brain. Glaucoma is commonly associated with increased sensitivity to intraocular pressure (IOP) and involves a proximal program that leads to RGC dendritic pruning and a distal program that underlies axonopathy in the optic projection. While genetic deletion of the Bcl2-associated X protein (*Bax*^-/-^) prolongs RGC body survival in models of glaucoma and optic nerve trauma, axonopathy persists, thus raising the question of whether dendrites and the RGC light response are protected. Here, we used an inducible model of glaucoma in *Bax*^-/-^ mice to determine if *Bax* contributes to RGC dendritic degeneration. We performed whole-cell recordings and dye filling in RGCs signaling light onset (αON-Sustained) and offset (αOFF-Sustained). We recovered RGC dendritic morphologies by confocal microscopy and analyzed dendritic arbor complexity and size. Additionally, we assessed RGC axon function by measuring anterograde axon transport of cholera toxin subunit B to the superior colliculus and behavioral spatial frequency threshold (i.e., spatial acuity). We found 1 month of IOP elevation did not cause significant RGC death in either WT or *Bax*^-/-^ retinas. However, IOP elevation reduced dendritic arbor complexity of WT αON-Sustained and αOFF-Sustained RGCs. In the absence of *Bax*, αON- and αOFF-Sustained RGC dendritic arbors remained intact following IOP elevation. In addition to dendrites, neuroprotection by *Bax*^-/-^ generalized to αON-and αOFF-Sustained RGC light- and current-evoked responses. Both anterograde axon transport and spatial acuity declined during IOP elevation in WT and *Bax*^-/-^ mice. Collectively, our results indicate *Bax* contributes to RGC dendritic degeneration and distinguishes the proximal and distal neurodegenerative programs involved during the progression of glaucoma.

**Supplementary Information:**

The online version contains supplementary material available at 10.1007/s12035-021-02675-5.

## Background

The BCL-2 (B-cell lymphoma-2) family of proteins contributes broadly to intrinsic, mitochondrial-based, apoptosis during development, cancer, and neurodegeneration [1]. During neurodegeneration, chronic cellular stress initiates apoptosis through the activation of Bcl-2-associated X (BAX) protein [1]. Upon activation, BAX proteins translocate from the cytosol to the mitochondrial outer membrane [2]. Within the mitochondrial outer membrane, BAX form heterodimers with BCL-2-antagonist/killer (BAK) or homodimers with other BAX proteins [3, 4]. Dimerization facilitates the formation of BAX oligomers [5] that permeabilize the mitochondrial outer membrane, causing the release of cytochrome *c* and second mitochondrial-derived activator of caspases (SMAC) into the cytosol, promoting cell death [6, 7].

Genetic deletion of *Bax* (*Bax*^-/-^) prevents cell death in affected tissues in models of neurodegeneration, including Parkinson’s disease [8], Alzheimer’s disease [9], amyotrophic lateral sclerosis [10], and glaucomatous optic neuropathy (glaucoma) [1, 11]. Glaucoma typically presents as an age-related neurodegenerative disease piqued by sensitivity of visual tissues to intraocular pressure (IOP). Glaucoma causes neurological blindness by targeting retinal ganglion cells (RGC) and their axons for degeneration through two main programs: a distal program in the optic nerve proper, affecting RGC axons, and a proximal program in the retina, affecting RGC somas, dendrites, and synapses [11–13]. Evidentiary support for compartmentalized neurodegeneration comes from inducible and chronic genetic models of glaucoma combined with gene or pharmaceutic dosing [13–20]. Neuroprotection of axons by administering nicotinamide and/or expressing the *slow Wallerian degeneration (Wld*^S^*)* allele reduces axon dropout, preserves anterograde axon transport, and protects spatial acuity, but somatic and dendritic degeneration persists [17–19, 21, 22]. *Bax*^-/-^ proves protective against RGC body death in models of glaucoma and acute focal injury to the optic nerve, yet axons comprising the optic nerve still undergo significant degeneration [14, 23].

Genetic and inducible models of glaucoma combined with genetic dosing of *Bax* and *Wld*^S^ elegantly demonstrate compartmentalized degeneration [11, 12, 18, 22]. Here, we sought to investigate the influence of *Bax*^-/-^ on RGC dendrites and signaling prior to significant cell death. Using an inducible microbead model of glaucoma [24], we found *Bax* contributes to dendritic pruning and degradation of evoked responses of alpha RGCs signaling light increments (αON-Sustained) and decrements (αOFF-Sustained). IOP elevation decreased anterograde axonal transport and spatial acuity during glaucoma in both WT and *Bax*^-/-^ animals. Our results indicate *Bax* contributes to the reduction in RGC dendritic complexity and signaling during IOP elevation and illustrates the separability of the proximal and distal programs involved in degeneration during glaucoma.

## Methods

### Animals and Genotype Confirmation

All experimental procedures were approved by the Vanderbilt University Institutional Animal Care and Use Committee. We obtained 6- to 8-week-old male C57Bl/6 (WT) mice from Charles River Laboratory (Wilmington, MA). We obtained 4- to 10-week-old male mice with a targeted null mutation in the *Bax* gene (*Bax*^-/-^, 002994 - B6.129X1-*Bax*^*tm1Sjk*^) from Jackson Laboratory (Bar Harbor, ME, [25]), We confirmed *Bax*^-/-^ by PCR according to the vendor’s protocol, using the following primers: GTT GAC CAG AGT GGC GTA GG (Common), CCG CTT CCA TTG CTC AGC GG (Mutant forward), and GAG CTG ATC AGA ACC ATC ATG (WT forward). We obtained primers from Integrated DNA Technologies (Coralville, IA). Mice were maintained on a 12-h light-dark cycle with standard rodent chow and water available as desired.

### IOP Elevation and Measurement

We anesthetized mice with isoflurane (2.5%), and we applied tropicamide (1%), proparacaine (0.5%), and lubricating drops to both eyes. We unilaterally elevated IOP by injecting 1.5 μL of 15-μm polystyrene microbeads (Invitrogen, Carlsbad, CA) into the anterior chamber of the eye via a borosilicate glass pipette attached to a micromanipulator (M3301R, WPI, Sarasota, FL) driven by a microsyringe pump (DMP, WPI, Sarasota, FL, [24]). For an internal control, we injected an equal volume of sterile phosphate-buffered saline (PBS) into the fellow eye. As previously described [24, 26–28], we measured IOP in anesthetized (2.5% isoflurane) mice by rebound tonometry (Tono-Pen XL, Reichert Technologies, Depew, NY). We measured baseline IOP of each eye 1 week before microbead/saline injection. After injections, we measured IOP at least 2 times per week for 1 month.

### Retinal Ganglion Cell Physiology

We performed whole-cell current-clamp (0 pA) recordings from RGCs in whole-mount retinas, using previously established protocols from our laboratory [27–29]. Whole retinas were removed from eyes and placed in a physiologic chamber. Retinas were continuously perfused at a rate of 2 mL/min with carbogen-saturated (95% O_2_, 5% CO_2_) bicarbonate-buffered Ames’ media supplemented with glucose (20 mM) heated to 32°C (Model TC-344C, Warner Instruments, Hamden, CT). Extracellular solution pH was 7.4 and osmolarity 290 (Vapro 5600, Wescor Inc., Logan, UT). Whole-cell recordings were performed using borosilicate pipettes filled with (in mM): 125 K-gluconate, 10 KCl, 10 HEPES, 10 EGTA, 4 Mg-ATP, 1 Na-GTP, and 0.1 ALEXA 555 (Invitrogen, Carlsbad, CA). Intracellular solution pH was 7.35 and osmolarity was 285. When filled with intracellular solution, pipette resistance was 4–8 MΩ. Electrical signals were amplified and sampled at ≥10 kHz (Multiclamp 700B, Digidata 1550A, Molecular Devices, San Jose, CA).

During whole-cell current-clamp recordings, we targeted alpha-type RGCs. For 70% of cells analyzed (256 out of 364), we determined the distance from the optic nerve head. On average, cells were in the mid peripheral retina. We tested RGC responses to light and current injections (0 to 180 pA in 20 pA increments). Light responses were evoked by a light-emitting diode system (pE-4000, CoolLED, Andover, UK), and light was focused onto the retina through the microscope objective (40×, full field, 365 nm, 3 s). After physiology, retinas were fixed overnight in 4% paraformaldehyde (PFA) at −4°C. The following day, the PFA solution was removed and substituted with PBS, and we prepared retinas for immunohistochemistry.

### Retinal Immunohistochemistry, Imaging, and Dendritic Morphologic Analysis

We performed immunohistochemistry on whole-mount retinas and sections from superior colliculus (SC) as previously described [26, 27, 29–31]. Retinas were immunolabeled with the following primary antibodies: mouse-non-phosphorylated neurofilament H (SMI-32,1:1000; 801701, BioLegend, San Diego, CA) and goat-choline acetyltransferase (ChAT, AB144P,1:100, Millipore, Burlington, MA). For a subset of experiments, we determined the density of RGCs in WT and *Bax*^-/-^ whole mount retinas by immunolabeling against mouse-SMI-32 (1:1000; 801701, BioLegend, San Diego, CA) and goat-Brn3a (1:200, Santa Cruz Biotechnology, Dallas, TX). For SMI-32-positive and Brn3a-positive cell counts, confocal micrographs were taken with an Olympus FV1000 using a 60× objective. Four images were taken within each quadrant (superior, inferior, nasal, temporal) of the retina. Cell counts were performed manually by a masked investigator and then averaged. SMI-32 intensity was determined by creating ROIs around the soma of each SMI-32-positive cell and normalizing to background. We determined neuronal density in SC sections by immunolabeling against rabbit-NeuN (1:500,12943, Cell Signaling Technology, Danvers, MA). Z-stack images were obtained using a 40× objective with a 2× zoom. Max intensity projections were generated for each z-stack and NeuN-positive cell counts were performed manually by a masked investigator.

We imaged tissues using confocal microscopy (Olympus FV-1000). RGC dendritic arbors were montaged and manually traced using Adobe Illustrator and Adobe Photoshop, respectively. Skeletonized dendritic arbors were analyzed using Fiji (ImageJ, version 1.53c). We determined the dendritic field area using the polygon selection tool in Fiji. We determined dendritic branch points by manually counting the number of dendritic bifurcations [22, 26]. Sholl analysis was performed using the Fiji plugin. For dendritic depth, we measured the distance from the start of the IPL to the inner most dendritic fluorescence signal. Six distance measurements were taken for each cell with regard to total IPL and dendritic depth, and then averaged. Dendritic depth was presented as a percentage of the relative total IPL depth for each cell.

### Anterograde Axonal Transport Analysis

Mice were anesthetized with isoflurane (2.5%), and we injected 1 μL of cholera toxin subunit B 488 (CTB-488, Molecular Probes, Eugene, OR) into the anterior chamber of both eyes [26, 27, 29, 32]. We allowed 2 days for transport of CTB-488 to the SC. After this time, we perfused mice transcardially with PBS followed by 4% PFA. After perfusion, we removed brains and cryoprotected them in sucrose (30%). We then obtained coronal midbrain sections (50 μm) using a freezing sliding microtome. After sectioning, alternating SC sections were mounted and imaged using a Nikon Ti Eclipse microscope (Nikon Instruments Inc., Melville, NY). We quantified the area of CTB-488 signal (intact transport) using a custom-written routine in ImagePro (Media Cybernetics, Bethesda, MD, [31]). Heat map surface plots show the fluorescence area relative to the total area of the SC. We confirmed successful CTB-488 uptake by RGCs in whole-mount retinas by confocal microscopy.

### Optic Nerve Transmission Electron Microscopy and Optic Nerve Axon Quantification

One month after IOP elevation, we perfused WT and *Bax*^-/-^ mice with 0.1 M cacodylate buffer followed by 2.5% glutaraldehyde in cacodylate buffer. We carefully removed eyes with the optic nerve attached. We then isolated 3-mm segments of optic nerve proximal to the globe and post-fixed them for 1 h in 2.5% glutaraldehyde in cacodylate buffer. Next, we embedded the optic nerve segments in Epon resin and obtained semi-thin (700 nm, light microscopy) and ultra-thin (70 nm, electron microscopy) cross-sections. We stained semi-thin cross-sections with 1% paraphenylenediamine (PPD; in a 1:1 mixture of methanol and 2-propanol) and 1% toluidine blue to identify myelin sheaths and glia, respectively. We imaged sections *en montage* using a Nikon H600L microscope equipped with a 100× oil-immersion objective, motorized X-Y-Z stage, a digital SLR camera, and differential interference contrast optics. Ultra-thin nerve cross-sections were prepared and photographed at 2700× (10 images per nerve for axon counts) and 240× magnification (one image per nerve to measure cross-sectional nerve area) using a Philips CM-12, 120-keV transmission electron microscope at Vanderbilt Cell Imaging Shared Resource Core. A naïve observer manually counted total and degenerating axons using Fiji ImageJ. We identified degenerating axons based on multilaminar myelin sheaths and diminishment of cytoskeletal content.

### Behavioral Spatial Frequency Threshold Measurement

We measured spatial frequency threshold (i.e., spatial acuity) using the OptoMotry system (Cerebral Mechanics Inc., Canada) as previously described [27, 29]. We determined spatial acuity by assessing the oculomotor reflex to drifting spatial frequency gratings at 100% contrast. Each spatial frequency was presented until a response (tracking) or no response (no tracking) was indicated by naïve experimenters. Mice were tested 3 times prior to microbead injection (baseline) and 2 times per week for 1 month after injections.

### Statistical Analysis

We analyzed data using GraphPad (Version 9, GraphPad Software Inc, San Diego, CA) or SigmaPlot (Version 12, Systat Software Inc., San Jose, CA). We determined if data fit a normal distribution using Shapiro-Wilk tests. If data passed normality tests, we performed parametric statistics; otherwise, non-parametric statistics were performed. We identify each statistical test performed in figure captions in the “Results” section.

## Results

### Bax^-/-^ Abates RGC Dendritic Loss During Glaucoma

**Fig. 1:**
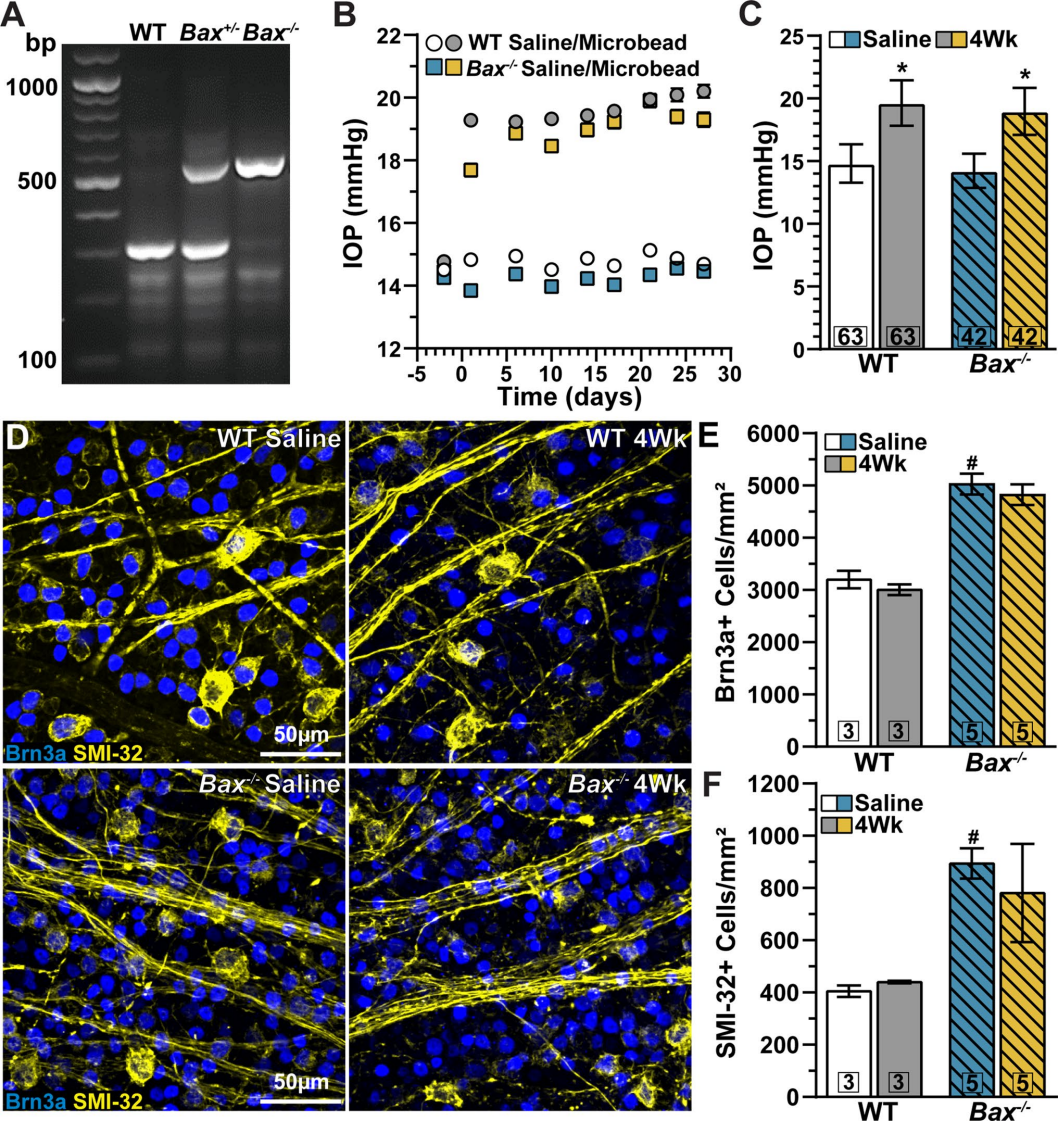
Genotype confirmation, IOP elevation, and RGC density. (A) PCR products of WT (304 bp), *Bax* heterozygote (*Bax*^+/-^; 304 bp and 507 bp) and *Bax* mutant (*Bax*^-/-^; 507 bp) mice. (B) Mean intraocular pressure (IOP) of WT (circles) and *Bax*^-/-^ (squares) mice before (day 0) and following (days ≥ 1) a single unilateral injection of polystyrene microbeadsor equivalent volume of saline. (C) Microbead injection significantly elevated IOP for 4 weeks in both WT and *Bax*^-/-^ mice: WT: 19.64 ± 1.82 vs. 14.81 ± 1.54 mmHg, *p*<0.001; *Bax*-/-: 18.97 ± 1.88 vs. 14.23 ± 1.36 mmHg, *p*<0.001). (D) Confocal micrographs of whole-mount retinas from saline- and microbead-injected eyes of WT (top) and *Bax*^-/-^ (bottom) mice immunolabeled against Brn3a (blue) and SMI-32 (yellow). (E–F) Brn3a+ and SMI-32+cell density was unaffected by 1 month of IOP elevation in both WT (p≥0.92) and *Bax*^-/-^ retinas (p≥0.83). *Bax*^-/-^ retinas contain significantly more Brn3a+ and SMI-32+ cells compared to WT (#, *p*≤0.01). Statistics: Student’s *t*-tests (C), one-way ANOVA, Tukey post hoc (E), Kruskal-Wallis test, Dunn’s post hoc (F). Data presented as mean ± SEM.

We verified the genotype of each *Bax* animal by gel electrophoresis of PCR products. Mice homozygous for the mutant *Bax*^tm1Sjk^ allele (*Bax*^-/-^) showed a single band at 507 bp. Heterozygous mice for the mutant *Bax*^tm1Sjk^ allele (*Bax*^*+/-*^) produced bands at 507 bp and 307, indicating the WT *Bax* allele. WT animals produced only a single band at 307 bp (Fig. 1A).

Before elevating intraocular pressure (IOP) by blocking the outflow of aqueous fluid by microbead occlusion of the trabecular meshwork, we measured IOP for 2 consecutive days (i.e., baseline). We did not detect a significant difference in baseline IOP between WT (14.64 ± 0.13 mmHg) and *Bax*^-/-^ naïve eyes (14.35 ± 0.16 mmHg, *p*=0.16, Fig. 1B). Following a single injection of microbeads into the anterior chamber of the eye, we achieved a sustained increase in IOP over the duration of the experiment (1 month) for both WT (+32.6%, 19.64 ± 1.82 vs. 14.81 ± 1.54 mmHg, *p*<0.001) and *Bax*^-/-^ eyes (+33.3%, 18.97 ± 1.88 vs. 14.23 ± 1.36 mmHg, *p*<0.001) compared to respective saline-injected eyes (Fig. 1B, C).

We recently reported elevating IOP for 1 month does not significantly affect the density of RBPMS-positive RGCs in WT retinas [22]. Again, we found IOP elevation does not significantly affect the density of Brn3a- (3242 ± 167 vs. 3046 ± 101 cells/mm^2^, *p*=0.92) or SMI-32-positive RGCs in WT retinas (413 ± 22 vs. 448 ± 5 cells/mm^2^, *p*>0.99, Fig. 1D–F). As previously observed [33], *Bax*^-/-^ retinas contain significantly more RGCs. Similarly, we found *Bax*^-/-^ significantly increases both Brn3a- (+56%, *p*=0.0002) and SMI-32-positive RGCs (+118%, *p*=0.01) compared to WTs (Fig. 1D–F**)**. However, IOP elevation does not significantly impact either Brn3a- (5066 ± 199 vs. 4863 ± 197 cells/mm^2^, *p*=0.839) or SMI-32-positive RGC density in *Bax*^-/-^ retinas (902 ± 58 vs. 789 ± 188 cells/mm^2^, *p*>0.99, Fig. 1D–F). Collectively, our results indicate modest IOP elevation for 1 month does not significantly affect RGC density in either WT or *Bax*^-/-^ retinas.

Although we find elevating IOP for 1 month does not cause significant RGC dropout, our result does not preclude the pro-degenerative impact of IOP elevation on RGC morphology and physiology. Our laboratory and others have found that RGC dendrites and voltage-gated responses are particularly sensitive to IOP elevation [22, 34–36]. Next, we determined if *Bax* contributes to the phenotypic dendritic pruning and response degradation observed in WT RGCs during IOP elevation.

**Fig. 2:**
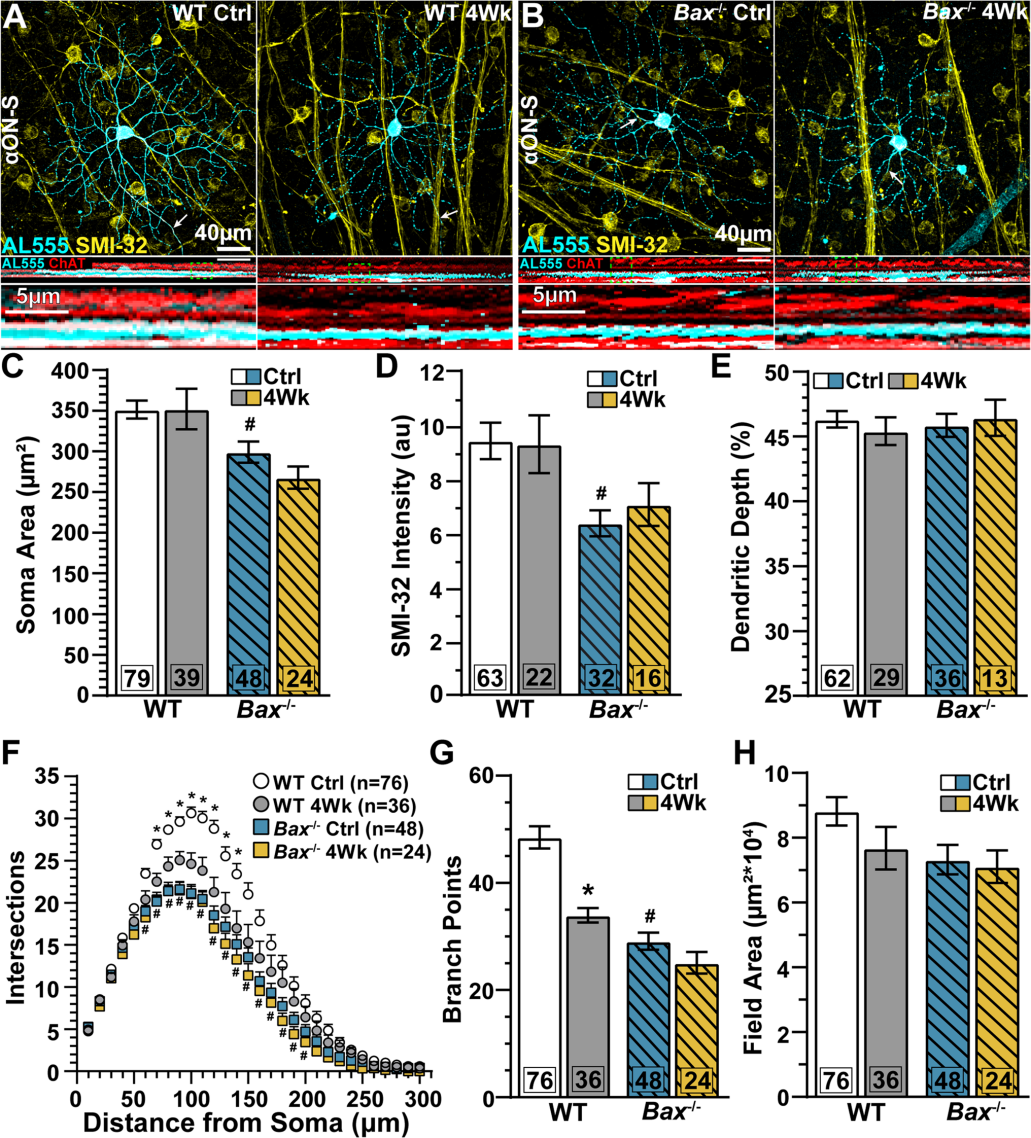
*Bax*^-/-^ reduces dendritic degeneration in αON-S RGCs during IOP elevation. (A–B) Confocal micrographs of αON-Sustained (αON-S) RGCs filled with ALEXA 555 (AL555, cyan) from saline- and microbead-injected eyes of (A) WT and (B) *Bax*^-/-^ animals. αON-S RGCs were neurochemically and morphologically identified by robust labeling against SMI-32 (yellow) and dendritic stratification within the ON sublamina of the inner plexiform layer (IPL), identified by ChAT (red). Arrows indicate RGC axons. Dashed green boxes indicate the RGC dendrites highlighted below. (C) IOP elevation did not affect soma size for either WT or *Bax*^-/-^ αON-S RGCs (*p*>0.99), but WT αON-S RGC somas are intrinsically larger than αON-S cell bodies from *Bax*^-/-^ retinas (#, *p*=0.02). (D) Regardless of genotype, elevating IOP did not significantly change SMI-32 immunolabeling intensity in αON-S RGCs (*p*≥0.13). Intensity of SMI-32 immunoreactivity is reduced in *Bax*^-/-^ αON-S RGCs (#, *p*=0.0006). (E) αON-S RGC dendritic depth was unaffected by IOP elevation or *Bax*^-/-^ (*p*≥0.87). (F) Sholl analysis revealed IOP elevation decreased αON-S RGC dendritic complexity inWTs (*, *p*≤0.04). *Bax*^-/-^ αON-S RGC dendritic arbors are intrinsically less complex compared to WTs (#, *p*≤0.03). (G) IOP elevation significantly reduced the number of branch points of WT αON-S RGCs (*, *p*<0.001), but *Bax*^-/-^ preserved αON-S RGCs branch points during IOP elevation (*p*<0.99). *Bax*^-/-^ αON-S RGCs have fewer branch points compared to WT cells (#, *p*<0.001). (H) Neither genotype nor IOP elevation affected dendritic field area (*p*≥0.23). The number of cells included in each dataset is indicated within each bar graph. The number of animals for each condition was: WT Ctrl *n*=60, WT 4Wk *n*=23, *Bax*^-/-^ Ctrl *n*=39, *Bax*^-/-^ 4Wk *n*=18. Statistics: Kruskal-Wallis test, Dunn’s post hoc (C, G, H), one-way ANOVA, Tukey post hoc (D, E), two-way repeated measures anova, Tukey’s post hoc (F). Data presented as mean ± SEM.

We identified αON-Sustained (αON-S) RGCs in WT and *Bax*^-/-^ retinas. WT (Fig. 2A) and *Bax*^-/-^ (Fig. 2B) αON-S RGCs have large somas with heavy immunolabeling for SMI-32 and their dendrites terminate within the “ON” sublayer of the inner plexiform layer (IPL). *Bax*^-/-^ intrinsically reduced soma area (*p*=0.02, Fig. 2C), SMI-32 immunoreactivity (*p*=0.0006, Fig. 2D), and dendritic complexity (Sholl analysis: *p*≤0.03; branch points: *p*<0.001, Fig. 2F, G) of αON-S RGCs compared to WTs. However, *Bax*^-/-^ does not inherently alter relative dendritic depth (Fig. 2E) or dendritic field area (Fig. 2H).

After 1 month of IOP elevation, we found a significant reduction in the number of dendritic intersections (Sholl analysis, *p*≤0.04, Fig. 2F) and a significant, 32%, decrease in branch points (48 ± 2 vs. 32.7 ± 1.4 branch points, *p*<0.001, Fig. 2G) in WT αON-S RGCs. We confirmed this reduction in dendritic branching was not due to sampling bias across retinal eccentricities by measuring the distance from the optic nerve head and comparing dendritic branching versus eccentricity. Typically, we recovered WT αON-S RGCs from the mid peripheral retina (WT Ctrl: 1485±59 μm, WT 4Wk: 1443±92 μm from the optic nerve head, *p*=0.98, Fig. S1A). Similar to an earlier report [37], we did not detect a significant linear relationship between branch points and retinal eccentricity for WT αON-S from control (*R*^2^=0.002, *p*=0.71) or microbead-injected eyes (*R*^2^=0.07, *p*=0.21, Fig. S1B).

*Bax*^-/-^ increases retinal area [38]. Consequently, during whole-cell recording and dye-filling, we often targeted RGCs that were more distant from the optic nerve head than WT RGCs and even beyond the extent of WT retinas (*p*=0.005, Fig. S1A). Following 1 month of IOP elevation, *Bax*^-/-^ αON-S RGC dendritic arbors remained intact based on the number of dendritic crossings (Sholl analysis, *p*≥0.11, Fig. 2F), the number of dendritic branch points (*p*>0.99, Fig. 2G), and dendritic field area (*p*>0.99, Fig. 2H). Similar to fellow WT cells, we did not detect a significant relationship between branch points and eccentricity of αON-S RGCs sampled from *Bax*^-/-^ control (*R*^2^=0.002, *p*=0.74) and microbead-injected eyes (*R*^2^=0.04, *p*=0.39, Fig. S1C). Overall, our findings indicate *Bax*^-/-^ attenuated the loss of αON-S RGC dendritic complexity during IOP elevation.

**Fig. 3:**
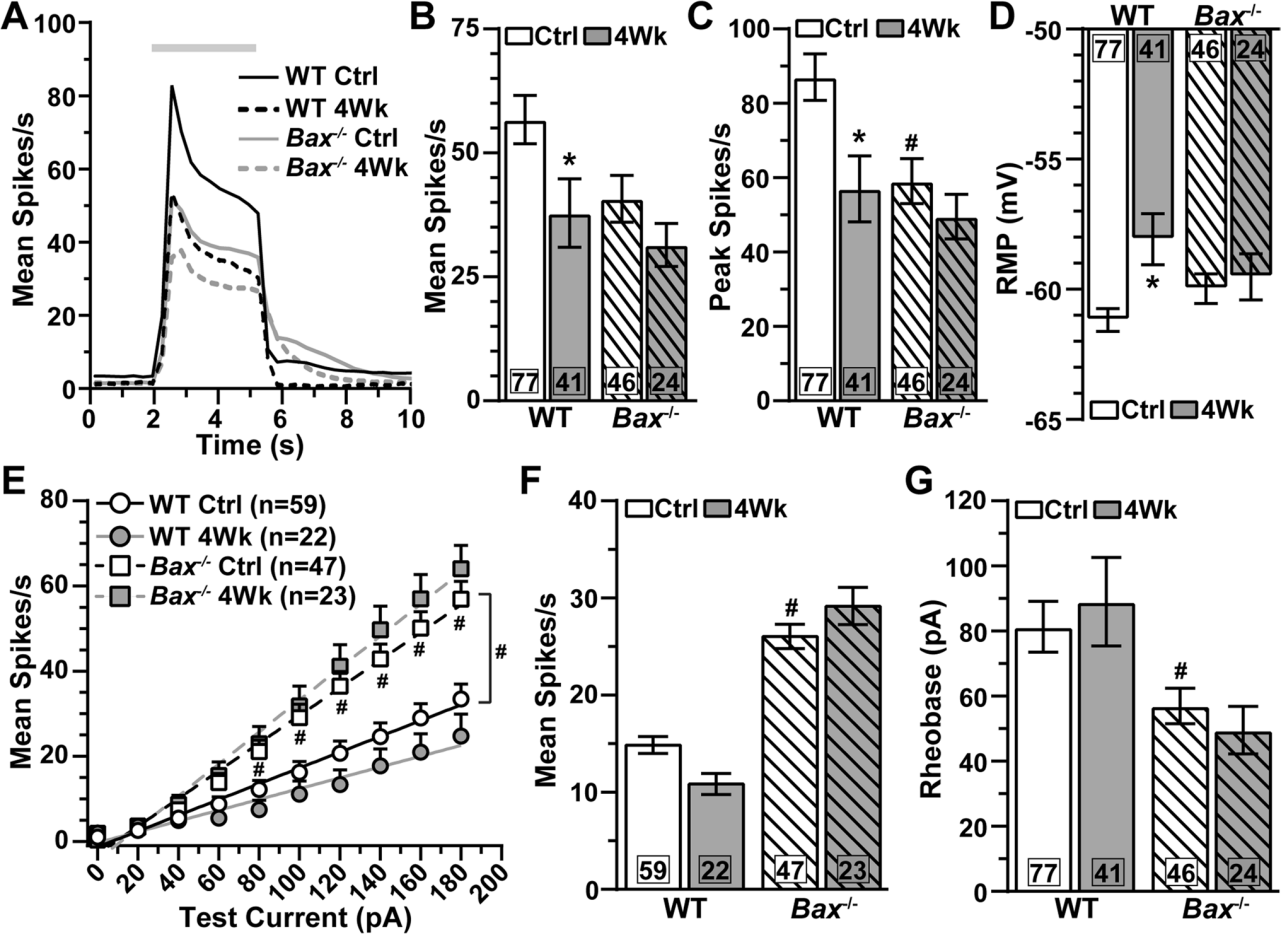
*Bax*^-/-^ protects light-evoked responses of αON-S RGCs during IOP elevation. (A) Mean spike rate (300 ms bin width) of αON-S RGCs from saline- and microbead-injected eyes of WT and *Bax*^-/-^ mice. Light duration (3 s) is indicated by the light gray horizontal line. (B–C) IOP elevation significantly diminished mean and peak light-evoked spike rate of WT αON-S RGCs (mean: *, *p*=0.004; peak: *, *p*=0.001), but *Bax*^-/-^ αON-S RGC light responses remained intact (*p*<0.99). *Bax*^-/-^ decreased light-evoked peak firing rate of αON-S RGCs compared to WT cells (#, *p*=0.04). (D) IOP elevation significantly depolarized resting membrane potential (RMP) of WT αON-S RGCs (*, *p*=0.003) but did not influence RMP in *Bax*^-/-^ αON-S RGCs (*p*=0.97). (E) Average spike rate of αON-S RGCs from saline- and microbead-injected eyes of WT and *Bax*^-/-^ mice to a series of depolarizing current injections (0 to 180 pA in 20 pA increments). IOP elevation did not significantly alter current-induced spike rate for either WT (*p*≥0.37) or *Bax*^-/-^ (*p*≥0.30) αON-S RGCs. *Bax*^-/-^ increased αON-S RGCs from control retinas current-evoked spiking compared to WT cells from control retinas (#, *p*≤0.01). Lines indicate best-fit linear regressions: WT Ctrl *R*^2^=0.25, WT 4Wk R^2^=0.21, *Bax*^-/-^ Ctrl *R*^2^=0.47, *Bax*^-/-^ 4Wk *R*^2^=0.55. The rate of change (slope) between spike rate and test current significantly increased for *Bax*^-/-^ control αON-S RGCs compared to WTs (#, *p*=0.0002). (F) *Bax*^-/-^ significantly increased spike activity averaged across current pulses (#, *p*<0.001). (G) One month of elevated IOP did not significantly affect WT or *Bax*^-/-^ αON-S rheobase (*p*≥0.41), but *Bax*^-/-^ intrinsically reduces rheobase (#, *p*=0.05). The number of cells included in each dataset is indicated within each bar graph. The number of animals for each condition was: WT Ctrl *n*=57, WT 4Wk *n*=24, *Bax*^-/-^ Ctrl *n*=39, *Bax*^-/-^ 4Wk *n*=18. Statistics: Kruskal-Wallis test, Dunn’s post hoc (B, C, F, G), one-way ANOVA, Tukey post hoc (D), two-way repeated measures ANOVA (E). Data presented as mean ± SEM.

In addition to morphological signatures, we identified αON-S RGCs based on response to light. αON-S RGCs from WT and *Bax*^-/-^ control eyes typically maintained low spontaneous activity in darkness and produced a robust train of action potentials for the duration of light onset (Fig. 3A). *Bax*^-/-^ rendered αON-S RGCs less responsive to light with regard to the mean (−28%, 56.7 ± 4.9 vs. 40.7 ± 4.7 spikes/s, *p*=0.36, Fig. 3B) and peak firing rate (−32%, 87 ± 6.2 vs. 59 ± 6 spikes/s, *p*=0.04, Fig. 3C). However, *Bax*^-/-^ does not affect αON-S RGC resting membrane potential (RMP, *p*=0.47, Fig. 3D). *Bax*^-/-^ intrinsically increased αON-S RGC responses to depolarizing current injections (*p*≤0.01, Fig. 3E, F) and decreased the amount of current required to modulate firing rate above baseline spiking (rheobase) by 30% (*p*=0.05, Fig. 3G).

In agreement with previous studies [22, 27, 36], we found 1 month of IOP elevation significantly reduced light-evoked mean (−33%, 56.7 ± 4.9 vs. 37.8 ± 6.9 spikes/s, *p*=0.004, Fig. 3B) and peak firing rate of WT αON-S RGCs (−34.5%, 87 ± 6.2 vs. 57 ± 8.9 spikes/s, *p*=0.001, Fig. 3C). Contrary to WT cells, light responses of αON-S RGCs from *Bax*^-/-^ microbead eyes are similar to respective control cells (*p*>0.99, Fig. 3B, C). IOP elevation significantly depolarized WT αON-S RGC RMP relative to control cells (+5%, −61 ± 0.44 vs. −58 ± 0.98 mV, *p*=0.003), but RMP of *Bax*^-/-^ αON-S cells appear unaffected by IOP elevation (*p*=0.97, Fig. 3D). After 1 month of IOP elevation, responses of αON-S RGCs to depolarizing current injections (*p*≥0.30, Fig. 3E, F) and rheobase (*p*≥0.41, Fig. 3G) are comparable to control cells independent of genotype.

**Fig. 4:**
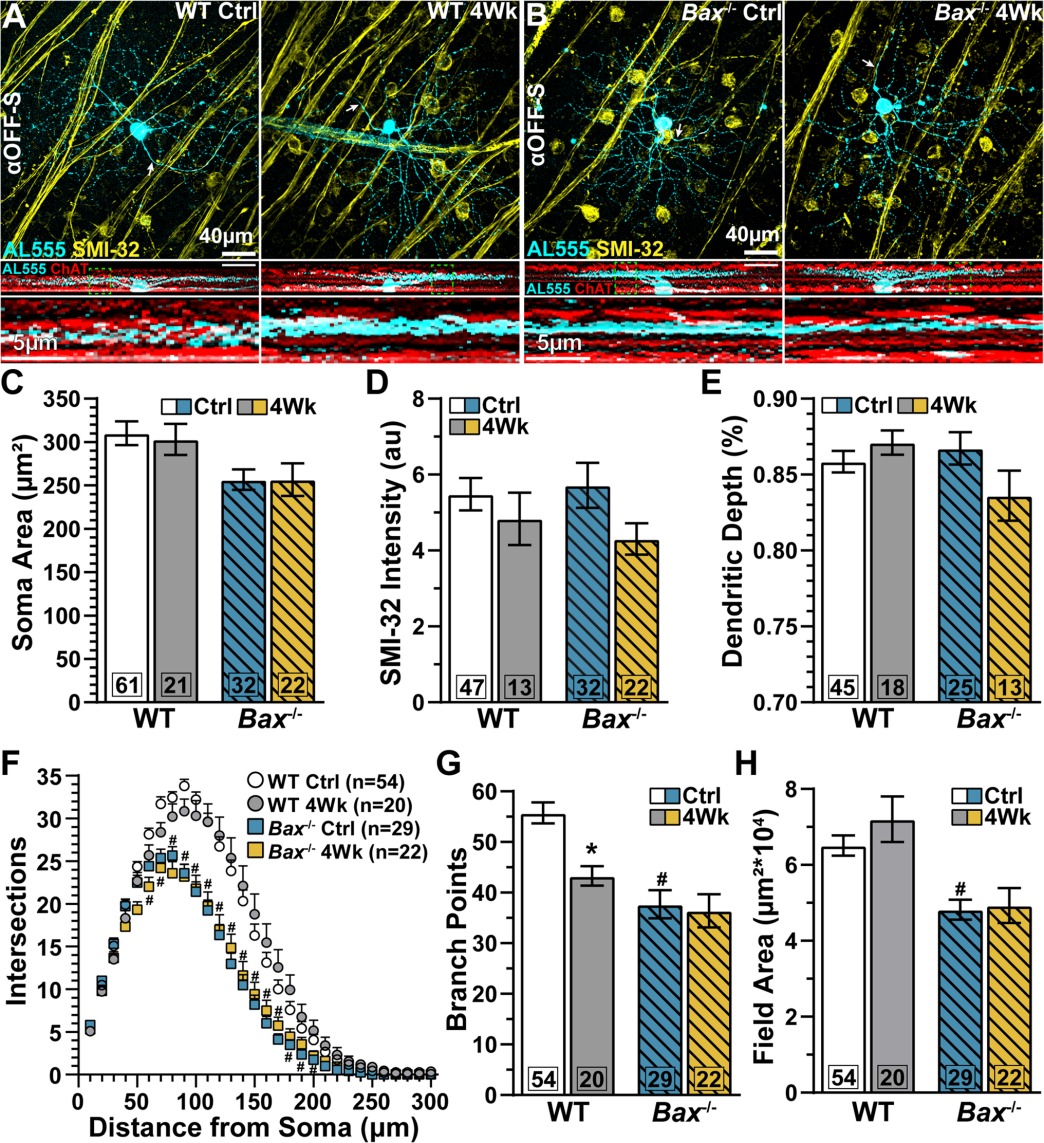
*Bax*^-/-^ protects αOFF-S RGC dendrites during IOP elevation. (A–B) Representative αOFF-S RGCs from whole-mount retinas of saline- and microbead-injected eyes of (A) WT and (B) *Bax*^-/-^ mice. RGCs were filled with ALEXA 555 (AL555, cyan) and morphologies were recovered by confocal microscopy. αOFF-S RGCs were neurochemically identified by modest immunoreactivity for SMI-32 (yellow) and morphologically characterized by dendritic arbor stratification within the distal OFF sublamina of the IPL defined by immunolabeling against ChAT (red). RGC axons indicated by arrows. Dashed green boxes show the dendritic ramifications highlighted below. (C–E) Neither IOP elevation nor *Bax*^-/-^ significantly impacted αOFF-S RGC (C) soma area (*p*≥0.13), (D) SMI-32 immunolabeling (*p*≥0.74), or (E) dendritic depth (*p*≥0.34). (F) Sholl analysis of WT and *Bax*^-/-^ αOFF-S dendritic intersections from saline- and microbead-injected eyes. IOP elevation did not significantly influence the number of dendritic crossings in WT (*p*≥0.09) or *Bax*^-/-^ αOFF-S RGCs (*p*≥0.07). *Bax*^-/-^ αOFF-S RGCs have fewer dendritic crossings compared to WTs (#, *p*≤0.04). (G) Elevating IOP significantly diminished the number of dendritic branch points of WT αOFF-S (*, *p*=0.02). *Bax*^-/-^ protects αOFF-S dendritic branch points during IOP elevation (*p*>0.99). The number of αOFF-S dendritic branch points is significantly reduced by *Bax*^-/-^ compared to WT controls (#, *p*<0.001). (H) Elevating IOP did not affect dendritic field area of αOFF-S RGCs of WT or *Bax*^-/-^ mice (*p*≥0.55). *Bax*^-/-^ significantly reduced αOFF-S RGC dendritic field area compared to WT controls (#, *p*=0.002). The number of cells included in each dataset is indicated within each bar graph. The number of animals for each condition was: WT Ctrl *n*=43, WT 4Wk *n*=18, *Bax*^-/-^ Ctrl *n*=27, *Bax*^-/-^ 4Wk *n*=16. Statistics: Kruskal-Wallis test, Dunn’s post hoc (C, D, E, G), two-way repeated measures ANOVA, Tukey’s post hoc (F), one-way ANOVA, Tukey post hoc (H). Data presented as mean ± SEM.

Next, we identified αOFF-Sustained (αOFF-S) RGCs in both WT (Fig. 4A) and *Bax*^-/-^ retinas (Fig. 4B) based on their large cell bodies, modest immunoreactivity to SMI-32, dendritic stratification just distal to the “OFF” sublamina of the IPL, and response to light [26, 27, 36]. Relative to WT control cells, *Bax*^-/-^ does not drastically alter αOFF-S RGC soma area (*p*=0.16, Fig. 4C), SMI-32 immunolabeling (*p*>0.99, Fig. 4D), or relative dendritic depth within the IPL (*p*>0.99, Fig. 4E). However, *Bax*^-/-^ significantly reduced the number of dendritic intersections (Sholl analysis, *p*≥0.04, Fig. 4F), branch points (*p*<0.001, Fig. 4G), and field area (*p*=0.002, Fig. 4H) of αOFF-S RGCs from control retinas compared to WT control cells of the same type.

Following 1 month of IOP elevation, the number of dendritic branching points in WT αOFF-S RGCs decreased by 22% (55.7 ± 2 vs. 43.3 ± 1.9 branch points, *p*=0.02, Fig. 4G). However, IOP elevation did not significantly affect dendritic branch points of αOFF-S RGC from *Bax*^-/-^ animals (37.7 ± 2.8 vs. 36.4 ± 3.3 branch points, *p*>0.99, Fig. 4G). We confirmed these findings by measuring the distance from the optic nerve head for most cells. We found αOFF-S RGCs from WT control (1545 ± 82 μm) and microbead-injected eyes (1598 ± 127 μm) similarly located in the mid peripheral retina (*p*=0.98, Fig. S1D), and so too were αOFF-S RGCs from *Bax*^*-/-*^ control (2168±113 μm) and microbead eyes (1934±184 μm, p=0.61, Fig. S1D). We did not detect a significant relationship between branch points and distance from optic nerve head of αOFF-S RGCs from WT control eyes (*R*^2^=0.06, *p*=0.09), but this relationship strengthened following IOP elevation (*R*^2^=0.3, *p*=0.01, Fig. S1E). We did not observe a significant relationship between dendritic branching and eccentricity of αOFF-S RGCs from *Bax*^-/-^ control (*R*^2^=0.002, *p*=0.82) or microbead-injected eyes (*R*^2^=0.09, *p*=0.26, Fig. S1F). Based on our results, *Bax* appears to promote dendritic pruning of αOFF-S RGCs during IOP elevation.

**Fig. 5:**
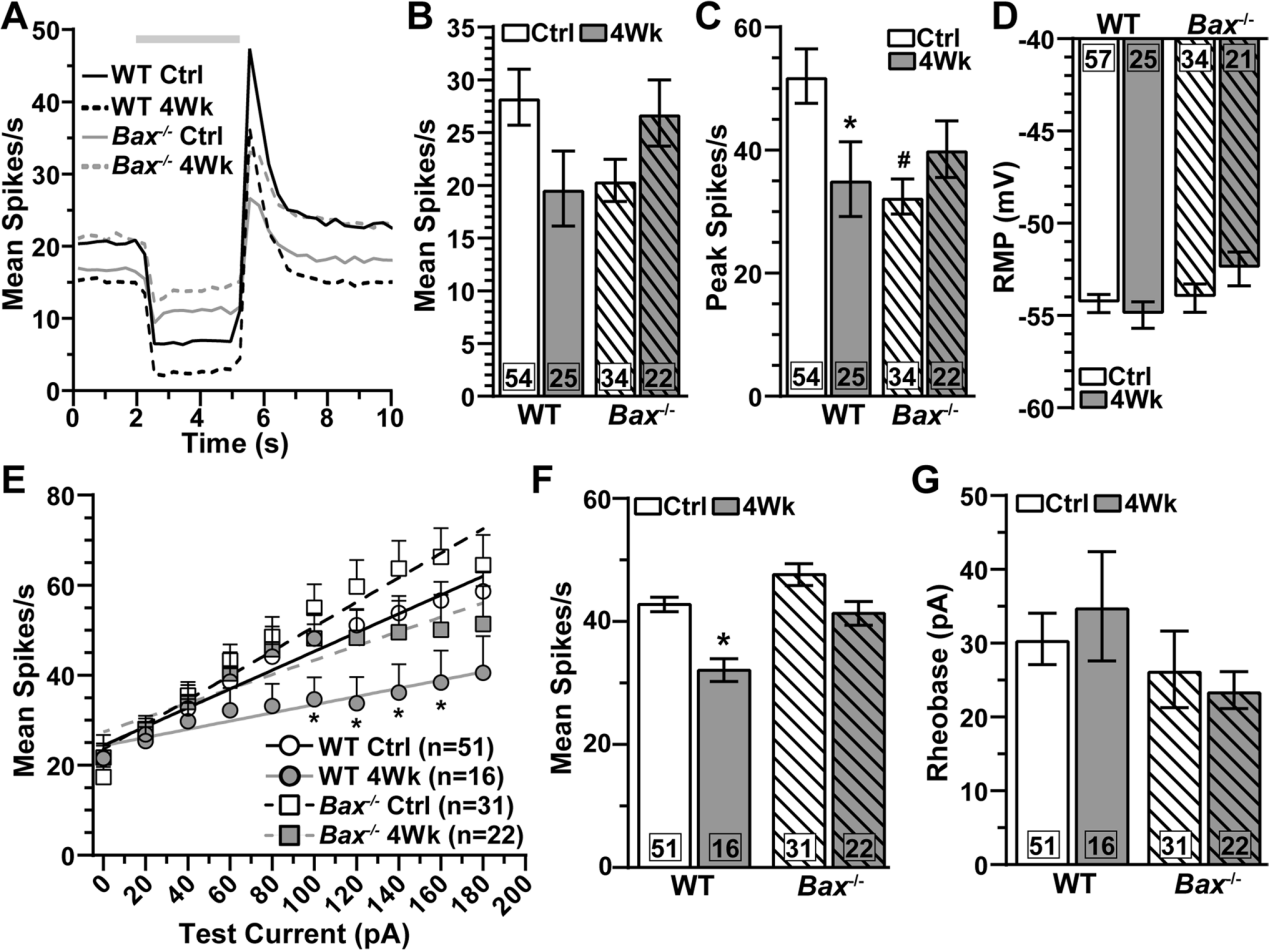
*Bax*^-/-^ protects light- and current-evoked responses of αOFF-S RGCs during glaucoma. (A) Light-evoked spike rate histograms of αOFF-S RGCs from saline- and microbead-injected eyes of WT and *Bax*^-/-^ animals. Light duration is indicated by the horizontal light gray line. (B) IOP elevation did not significantly affect the mean light-evoked spike rate in either WT (*p*=0.13) or *Bax*^-/-^ αOFF-S RGCs (*p*=0.90). (C) Elevating IOP significantly blunted the light-induced peak firing of WT αOFF-S RGCs (*, *p*=0.05), but *Bax*^-/-^ protects peak spike rate in these cells (*p*=0.73). *Bax*^-/-^ reduced peak firing rate of αOFF-S RGCs (#, *p*=0.007). (D) After 1 month of IOP elevation, RMP of αOFF-S RGCs is similar to respective control cells (*p*≥0.43). (E) IOP elevation significantly reduced current-evoked spike rate of WT αOFF-S RGCs (*, *p*≤0.03). Lines represent best-fit linear regressions. WT Ctrl *R*^2^=0.20, WT 4Wk *R*^2^=0.05, *Bax*^-/-^ Ctrl *R*^2^=0.25, *Bax*^-/-^ 4Wk *R*^2^=0.10. (F) IOP elevation significantly decreased spike activity of WT αOFF-S RGCs when averaged across current pulses (*, *p*<0.001) (G) Following 4 weeks of IOP elevation, WT and *Bax*^-/-^ αOFF-S RGC rheobase is similar to respective control cells (*p*≥0.08). The number of cells included in each dataset is indicated within each bar graph. The number of animals for each condition was WT Ctrl *n*=39, WT 4Wk *n*=19, *Bax*^-/-^ Ctrl *n*=27, *Bax*^-/-^ 4Wk *n*=16. Statistics: Kruskal-Wallis test, Dunn’s post hoc (B, D, F, G), one-way ANOVA, Tukey post hoc (C), two-way repeated measures ANOVA. Two-stage linear step-up post hoc (E). Data presented as mean ± SEM.

In addition to morphological features, we also identified αOFF-S RGCs by their physiologic responses [26, 27, 35, 36]. αOFF-S RGCs from both WT and *Bax*^-/-^ mice produced spontaneous action potentials during darkness, which were suppressed during light onset, and generated a sustained burst of action potentials after light offset (Fig. 5A). αOFF-S RGC light responses from *Bax*^-/-^ control eyes were generally smaller than their WT counterparts (Mean Spike Rate: 28.36 ± 2.65 vs. 20.48 ± 2 spikes/s, *p*=0.52; Peak Spike Rate: 52.1 ± 4.4 vs. 32.4 ± 2.8 spikes/s, *p*=0.007, Fig. 5B, C). *Bax*^-/-^ did not significantly affect RMP of αOFF-S RGCs from control eyes (*p*>0.99, Fig. 5D). Contrary to light responses, when we stimulated WT and *Bax*^-/-^ control αOFF-S RGCs with depolarizing current injections, we found mean spike rate (*p*≥0.17, Fig. 5E, F) and rheobase (*p*=0.12, Fig. 5G) to be similar.

One month of IOP elevation reduced the mean (28.4 ± 2.65 vs.19.7 ± 3.56 spikes/s, *p*=0.13, Fig. 5B) and peak firing rate at light offset of WT αOFF-S RGCs (52.1 ± 4.4 vs. 35.3 ± 6 spikes/s, *p*=0.05, Fig. 5C). *Bax*^-/-^ prevented this IOP-induced reduction in light-evoked mean (*p*=0.91) and peak spike rate (*p*=0.73) in αOFF-S RGCs (Fig. 5B, C). *Bax*^-/-^ does not appear to protect firing rate through mechanisms generating RMP because RMP of αOFF-S from control and microbead eyes is similar independent of genotype (*p*=0.12, Fig. 5D).

Next, we tested the impact of IOP elevation on αOFF-S RGC responses to a series of depolarizing current injections. Like the reduction in light responses, 1 month of IOP elevation decreased spike rate at suprathreshold test currents in WT αOFF-S RGCs (*p*≥0.03, Fig. 3E), and significantly reduced the average spike rate to depolarizing currents (*p*=0.001, Fig. 5F). However, IOP elevation does not significantly affect *Bax*^-/-^ αOFF-S RGC responses to depolarizing currents (*p*≥0.11, Fig. 5E, F) or rheobase (*p*>0.99, Fig. 5G). Taken together, our results show IOP elevation significantly alters WT RGC morphology and physiology regardless of cell type, and this pathology is largely undetectable in the absence of *Bax*.

### Axonopathy Endures in Bax^-/-^ Mice

**Fig. 6:**
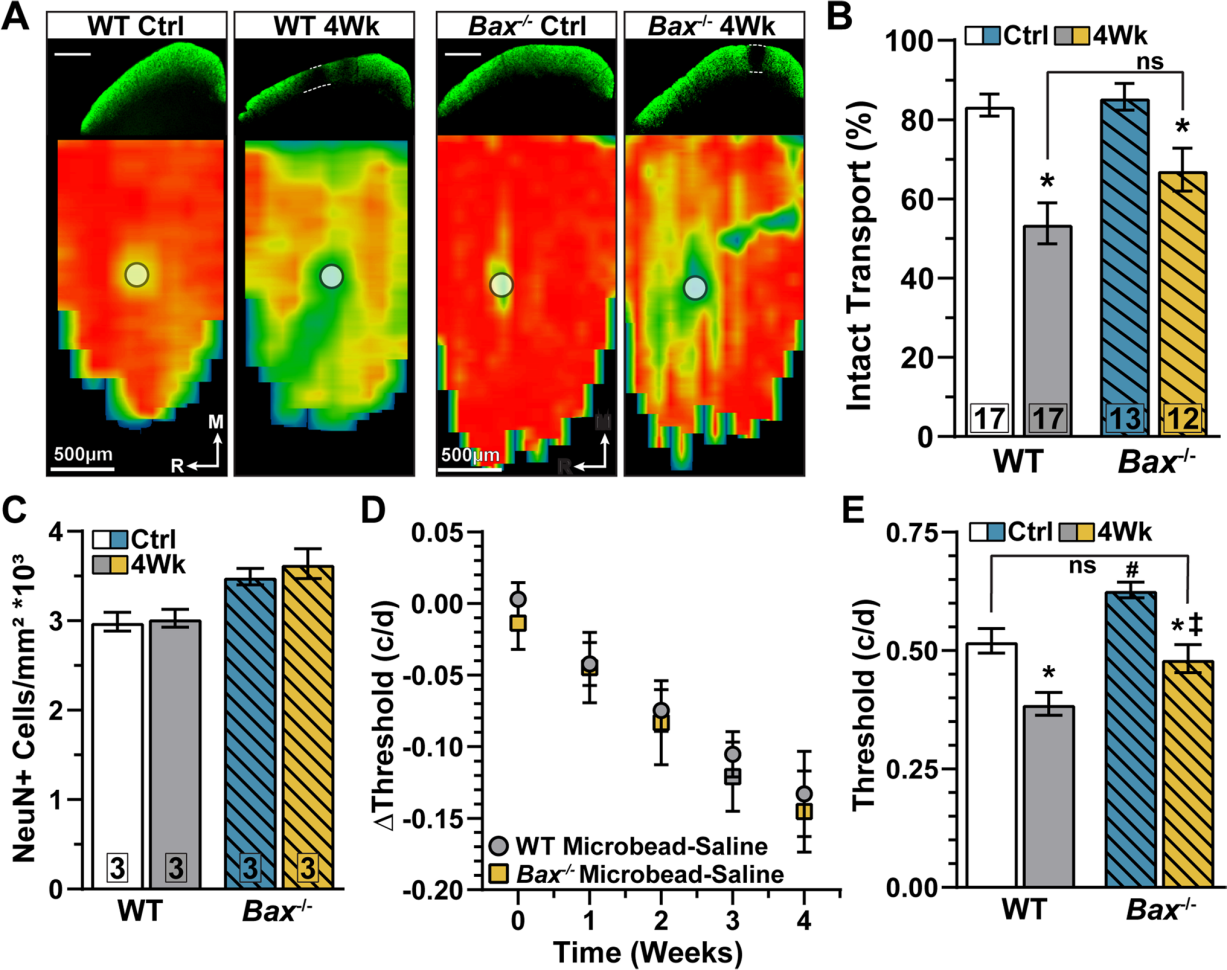
IOP elevation reduces anterograde axonal transport and spatial acuity. (A, top) Representative coronal sections of the superior colliculus (SC) after intravitreal injection of CTB (green) into WT (left) and *Bax*^-/-^ (right) control and 4-week microbead eyes. Scale bar = 500 μm. Dotted lines indicate deficits in CTB transport. (A, bottom) Example retinotopic heatmaps reconstructed from CTB intensity measurements in SC sections. Optic discs are indicated by filled white circles. Medial (M) and rostral (R) orientations are indicated. (B) IOP elevation significantly reduced anterograde axon transport of CTB in both WT (*p*=0.0002) and *Bax*^-/-^ mice (*p*=0.03). (C) Elevating IOP did not significantly affect the density of NeuN+ cells in the SC of WT or *Bax*^-/-^ mice (*p*≥0.82). (D) Spatial frequency threshold (cycles/degree, c/d) of microbead minus saline injected eyes (ΔThreshold) from WT and *Bax*^-/-^ mice 1 to 4 weeks post injection (*p*>0.99). (E) *Bax*^-/-^ significantly increased spatial frequency resolution (#, *p*≤0.03). Four weeks of IOP elevation significantly reduced spatial frequency threshold in WT (*, *p*=0.0009) and *Bax*^-/-^ mice (*, *p*=0.004). However, spatial acuity of *Bax*^-/-^ microbead-injected eyes remained better than microbead-injected eyes of WT mice (ǂ, *p*=0.05) and not significantly different compared to saline-injected eyes of WTs (*p*=0.73). Spatial frequency threshold animal numbers: baseline (time 0): WT *n*=55, *Bax*^-/-^ *n*=60; 1 week: WT *n*=37, *Bax*^-/-^ *n*=40; 2 weeks: WT *n*=44, *Bax*^-/-^ *n*=40; 3 weeks: WT *n*=44, *Bax*^-/-^ *n*=40; 4 weeks: WT *n*=20, *Bax*^-/-^ *n*=13. Statistics: Kruskal-Wallis test, Dunn’s post hoc (B, E), one-way ANOVA, Tukey post hoc (C), two-way repeated measures ANOVA, Tukey’s post hoc (D). Data presented as mean ± SEM.

Degradation of RGC anterograde axon transport to the superior colliculus (SC) is an early indicator of axonopathy in glaucoma [31, 39]. Anterograde axon transport deficits occur prior to optic nerve axon degeneration and loss of postsynaptic target neurons of the SC [31, 39]. Based on this premise, we investigated anterograde axon transport of cholera toxin subunit B (CTB-488) to the SC. Following 1 month of IOP elevation, we observed a reduction in intact anterograde transport of CTB-488 to the SC in both WT and *Bax*^-/-^ mice (Fig. 6A). When quantified, we found IOP elevation significantly diminished the percent of intact transport in WT (−35%, *p*=0.0002) and *Bax*^-/-^ mice (−21%, p=0.03, Fig. 6B). We did not observe a statistical difference in the percentage of intact transport in the SC of WT and *Bax*^-/-^ mice subjected to IOP elevation (*p*=0.95, Fig. 6B).

The IOP-induced loss of anterograde axon transport to the SC does not appear to be due to outright degeneration of RGC axons in the optic nerve proper or SC neurons. As noted by others, and confirmed here, *Bax*^-/-^ intrinsically increased optic nerve size and axon density (Fig. S2A, B). When quantified, we found *Bax*^-/-^ significantly increased optic nerve cross-sectional area by 59% (60420 ± 4975 vs. 96308 ± 11607 μm^2^, *p*=0.009, Fig. S2E). As a corollary, *Bax*^-/-^ also significantly increased optic nerve axon density (+56%, 0.55 ± 0.05 vs. 0.87 ± 0.02 axons/μm^2^, *p*=0.0005, Fig. S2F). We observed no significant difference in the percent of degenerating axons between WT (2.94%) and *Bax*^-/-^ (2.12%) optic nerves from control eyes (*p*=0.85, Fig. S2G). Following 1 month of IOP elevation, we found optic nerve cross-sectional area (*p*≥0.98), axon density (*p*≥0.17), and the percentage of degenerating axons (*p*≥0.76) largely unchanged in both WT and *Bax*^-/-^ mice (Fig. S2E-G). The increase in RGC axon density observed in *Bax*^-/-^ optic nerves is supported by a modest increase in SC neuron density (*p*=0.09, Fig. 6C), but we did not detect a significant effect of IOP elevation on NeuN-positive cell density in the SC of either WT or *Bax*^-/-^ animals (*p*≥0.82, Fig. 6C).

We recently reported 1 month of IOP elevation worsens spatial frequency threshold (spatial acuity) in WT mice [22]. Here, we determined the influence of *Bax* on spatial acuity when challenged by IOP elevation. We measured the optomotor response to a series of 100% contrast sinusoidal gratings varying by spatial frequency. We found spatial acuity is similarly reduced for both WT and *Bax*^-/-^ mice over the course of IOP elevation (*p*>0.99, Fig. 6D).

Interestingly, *Bax*^-/-^ intrinsically *improved* spatial acuity by 21% compared to WT control eyes (0.52 ± 0.025 vs. 0.63 ± 0.016 c/d, *p*<0.001, Fig. 6E). Moreover, we found spatial acuity of *Bax*^-/-^ microbead-injected eyes is modestly better than microbead-injected eyes of WT mice (+24.8%, *p*=0.05) and not significantly different compared to saline-injected eyes of WTs (*p*=0.73, Fig. 6E). Following 1 month of IOP elevation, spatial acuity significantly decreased in the microbead-injected eyes of both WT (−25.5%, *p*=0.0009) and *Bax*^-/-^ (−23.8%, *p*=0.004) animals compared to respective control eyes (Fig. 6E).

## Discussion

Our results confirm and extend the evidence that *Bax*^-/-^ profoundly effects RGC somato-dendritic and axonal morphology [33, 40]. *Bax* deficiency increases RGC density (Fig. 1D–F), decreases RGC soma size and dendritic arbor complexity (Fig. 2C, F, G; Fig. 4C, F, G), and increases optic nerve area and RGC axon density (Fig. S2). Despite these gross morphological changes, alpha-type RGCs retain some semblance of their molecular (Figs. 1D, 2D, and 4D), morphological (Figs. 2E and 4E), and physiologic identity (Fig. 3A–D; Fig. 5A–D).

We found 1 month of IOP elevation does not cause significant RGC body degeneration in either WT or *Bax*^-/-^ retinas (Fig. 1D-F). Similarly, we have previously found IOP elevation by microbead occlusion of the trabecular meshwork for 1 month does not cause significant RGC body dropout [22, 41]. Although 1 month of IOP elevation does not produce significant RGC body elimination [22, 41], RGC dendritic arbor complexity diminishes in the presence of *Bax* [22, 27, 36]. Here, we found this to be true for both αON-S (Fig. 2G) and αOFF-S RGCs from WT retinas (Fig. 4G). *Bax*^-/-^ reduces dendritic pruning of αRGCs during IOP elevation (Figs. 2G and 4G).

How does *Bax*^-/-^ protect RGC dendrites against IOP-induced degeneration? In the absence of *Bax*, RGCs may be less vulnerable to injury because cells are inherently smaller and less complex (Figs. 2 and 4). Previously, we have found evidence lending support to this idea. Mutation in the transient receptor potential vanilloid 1 (TRPV1) channel, which signal stress through calcium conductance, also intrinsically reduces αRGC dendritic complexity, and the dendrites of these very cells are less vulnerable to degeneration during IOP elevation [26]. However, the possibly also exists that in the absence of stress-induced TRPV1-mediated calcium signaling, dendrites are less susceptible to degeneration. To overcome this problem of determining the influence of intrinsic developmental versus mechanistic changes on outcome measurements, which is typical when using conventional genetic models, inducible genetic constructs may be employed [42].

An alternative and mechanistic hypothesis for dendritic degeneration in WT RGCs is BAX also serves non-apoptotic functions that contribute to dendritic pruning [23, 43–46]. Local short-term activation of BAX/caspase-3 causes loss of excitatory surface receptors and dendritic pruning in the absence of apoptosis [42, 43, 47, 48]. Like the non-apoptotic actions of BAX, the complement pathway contributes to sculpting dendritic arbors and synapses following injury [13, 42, 43, 49, 50]. During DBA2/J glaucoma, complement protein C1qa increasingly colocalizes to PSD-95 within RGC dendrites, marking synapses for elimination [50]. Genetic deletion of *C1qa* or pharmacological antagonism of complement component, C1, reduces dendritic degeneration during glaucoma [13]. Taken together, upregulation of C1q might promote the activation of BAX during stress, leading to dendritic pruning in RGCs [51].

*Bax*^-/-^ also appears to protect light- and current-evoked responses of αON-S and αOFF-S RGCs during IOP elevation (Figs. 3 and 5). As we have previously reported [22, 27], and confirm here, 1 month of IOP elevation significantly reduced light-evoked responses of WT αON-S (Fig. 3A–C) and αOFF-S RGCs (Fig. 5A–C). Similarly, IOP elevation modestly reduced responses of αON-S RGCs to current stimulation (Fig. 3E, F) and significantly diminished current-evoked responses of αOFF-S RGCs (Fig. 5E, F). However, we found light- and current-evoked responses of αON-S (Fig. 3E, F) and αOFF-S RGCs (Fig. 5E, F) from *Bax*^-/-^ retinas resistant to degradation caused by IOP elevation.

*Bax* appears to contribute to the IOP-induced reduction in light- and current-evoked responses of WT RGCs (Figs. 3 and 5). This claim is supported by evidence showing that modest activation of BAX/caspase-3 is required for the induction of NMDA receptor-mediated long-term depression (LTD) through the endocytosis of AMPA receptors [42]. As a corollary, *Bax* knockdown inhibits induction of LTD [42]. Interestingly, not only do we find *Bax*^-/-^ protects light- and current-evoked responses during IOP elevation, but the current-evoked responses of αON-S RGCs from *Bax*^-/-^ control eyes are significantly larger and rheobase smaller compared to WTs (Fig. 3E–G). This enhancement in current-evoked activity of αON-S RGCs from *Bax*^-/-^ retinas may be due to a lack of LTD. Unlike WT αON-S RGCs, current-evoked responses from *Bax*^-/-^ αOFF-S RGCs are like those from WT retinas (Fig. 5E–G). In agreement with these divergent findings, only αON-S RGCs utilize NMDA receptor–mediated synaptic plasticity [52]. In regard to αOFF-S RGCs, we predict responses decline during stress due to increased metabolic burden to support electrogenic mechanisms [22]. In respect to metabolism, *Bax*^-/-^ may also support physiologic responses of αOFF-S RGCs, in particular, by protecting mitochondrial respiration and decreasing the amount of reactive oxygen species caused by nerve growth factor deprivation during stress [53–57].

As noted above, we found current-evoked responses of αON-S and αOFF-S RGCs from *Bax*^-/-^ control eyes are similar to or exceeded responses of their WT counterparts (Fig. 3E, F; Fig. 5 E, F). This finding is unsurprising given that smaller cells are typically more excitable [58]. However, the light responses of αON-S and αOFF-S RGCs from *Bax*^-/-^ control eyes were typically smaller compared to respective cell types from WT control eyes. This result could be explained by a decrease in excitatory input. This idea is supported by our finding that *Bax*^-/-^ reduces dendritic field complexity for both αON-S (Fig. 2F, G) and αOFF-S RGCs (Fig. 4F, G). Similarly, *Bax*^-/-^ reduces dendritic field area of VGluT3-positive amacrine and type 7 cone bipolar cells [59, 60]. Although *Bax*^-/-^ decreases bipolar cell dendritic field area, the number of synapses with cone axons is similar to WT [59]. In support of these anatomical data, we find spatial acuity is naturally increased in control eyes of *Bax*^-/-^ mice (Fig. 6E). These data indicate, *Bax*^-/-^ increases spatial resolution at the expense of light sensitivity.

Finally, we found 1 month of IOP elevation significantly reduces anterograde axon transport (Fig. 6A, B) and spatial acuity (Fig. 6D, E) in WT and *Bax*^-/-^ mice. Axon transport and spatial acuity deficits occurred due to IOP elevation and prior to outright RGC body (Fig. 1D–F) and axon degeneration (Fig. S2). Our findings extend the evidence that axonopathy endures not only during DBA2/J glaucoma or acute injury to the optic nerve but also in induced glaucoma in the absence of *Bax* [14, 18, 23]. The neuroprotective influence of *Bax*^-/-^ on RGC somas [14, 18, 23] and dendrites (Figs. 2 and 4) concurrent with axonopathy (Fig. 6A, B), reinforces the separability of the proximal and distal programs for neurodegeneration during glaucoma [11, 12].

## Conclusions

Our results indicate that *Bax* contributes to RGC dendritic degeneration and response degradation during glaucoma. In WT mice, 1 month of IOP elevation caused significant dendritic pruning and decline in responses to light in both αON-S and αOFF-S RGCs. *Bax*^-/-^ robustly reduced dendritic pruning and stabilized light signaling in αON-S and αOFF-S RGCs during IOP elevation. In agreement with previous studies, we found axonopathy endures during glaucoma in the absence of *Bax*.

## Supplementary Information

Figure S1. **αRGC Branch Points Versus Eccentricity.** (**A**) Distance from optic nerve head (ONH) of αON-S RGCs from control and microbead-injected eyes of WT and *Bax*^-/-^ mice. WT αON-S RGCs from control and microbead-injected eyes were sampled at similar retinal eccentricities (p=0.98). αON-S RGCs from *Bax*^-/-^ animals were sampled at more distant locations compared to WTs (#, p=0.0005). *Bax*^-/-^ αON-S RGCs from control and microbead-injected eyes were sampled at similar retinal eccentricities (p=0.36). (**B-C**) Relationship between branch points and distance from ONH of αON-S RGCs from WT (**B**) and *Bax*^-/-^ (**C**) control and microbead-injected eyes. (**D**) Distance from ONH of αOFF-S RGCs from control and microbead-injected eyes of WT and *Bax*^-/-^ mice. WT αOFF-S RGCs from control and microbead-injected eyes were sampled at comparable retinal eccentricities (p=0.98). αOFF-S RGCs from *Bax*^-/-^ animals were sampled at more distant eccentricities compared to WT cells (#, p=0.0002). *Bax*^-/-^ αOFF-S RGCs from control and microbead-injected eyes were sampled at similar distances (p=0.61). (**E-F**) Relationship between branch points and distance from ONH of αOFF-S RGCs from WT (**E**) and *Bax*^-/-^ (**F**) control and microbead-injected eyes. Statistics: One-way ANOVA Tukey Post hoc test (**A, D**), Linear regression (**B, C, E, F**). Bar graphs indicate mean, Error bars indicate SEM. Sample number is indicated within bars. (PNG 298 kb)

High resolution image (TIF 1670 kb)

Figure S2. **IOP Elevation Does Not Affect Optic Nerve Area or RGC Axon Density.** (**A-B**) Representative semi-thin cross-sections of optic nerves from WT (**A**) and *Bax*^-/-^ (**B**) control and microbead-injected eyes. Example ultra-thin cross-section of optic nerve from WT (**C**) and *Bax*^-/-^ (**D**) control and microbead-injected eyes. Example degenerating axons exhibiting multilaminar myelin sheaths indicated by arrowheads. (**E**) IOP elevation does not affect nerve area (p≥0.98), (**F**) axon density (p≥0.175), or (**G**) percent of degenerating axons (p≥0.767) in either WT or *Bax*^-/-^ nerves. However, *Bax*^-/-^ significantly increases nerve area (p=0.009) and axon density (p=0.0005). Statistics: One-way ANOVA Tukey Post hoc test (**C**). Bar graphs indicate mean, Error bars indicate SEM. Animal numbers: WT Ctrl n = 5, WT 4Wk n = 4, *Bax*^-/-^ Ctrl n = 4, *Bax*^-/-^ 4Wk n = 4. (PNG 2684 kb)

High resolution image (TIF 9261 kb)

## Abbreviations

RGC: Retinal ganglion cells
IOP: Intraocular pressure
WT: Wild type
Bcl-2: B-cell lymphoma 2
Bax: Bcl-2-associated X protein
Brn3a: Pou domain, class 4, transcription factor 1
SMI-32: Non-phosphorylated neurofilament H
RMP: Resting membrane potential
IPL: Inner plexiform layer
CTB: Cholera toxin subunit B
ChAT: Choline acetyltransferase
SC: Superior colliculus
