# Supplementary figures and images for: Bax Contributes to Retinal Ganglion Cell Dendritic Degeneration During Glaucoma
Source: Mol Neurobiol. 2022 Jan 5;59(3):1366–80. doi: 10.1007/s12035-021-02675-5 (PMC8882107; doi:10.1007/s12035-021-02675-5)

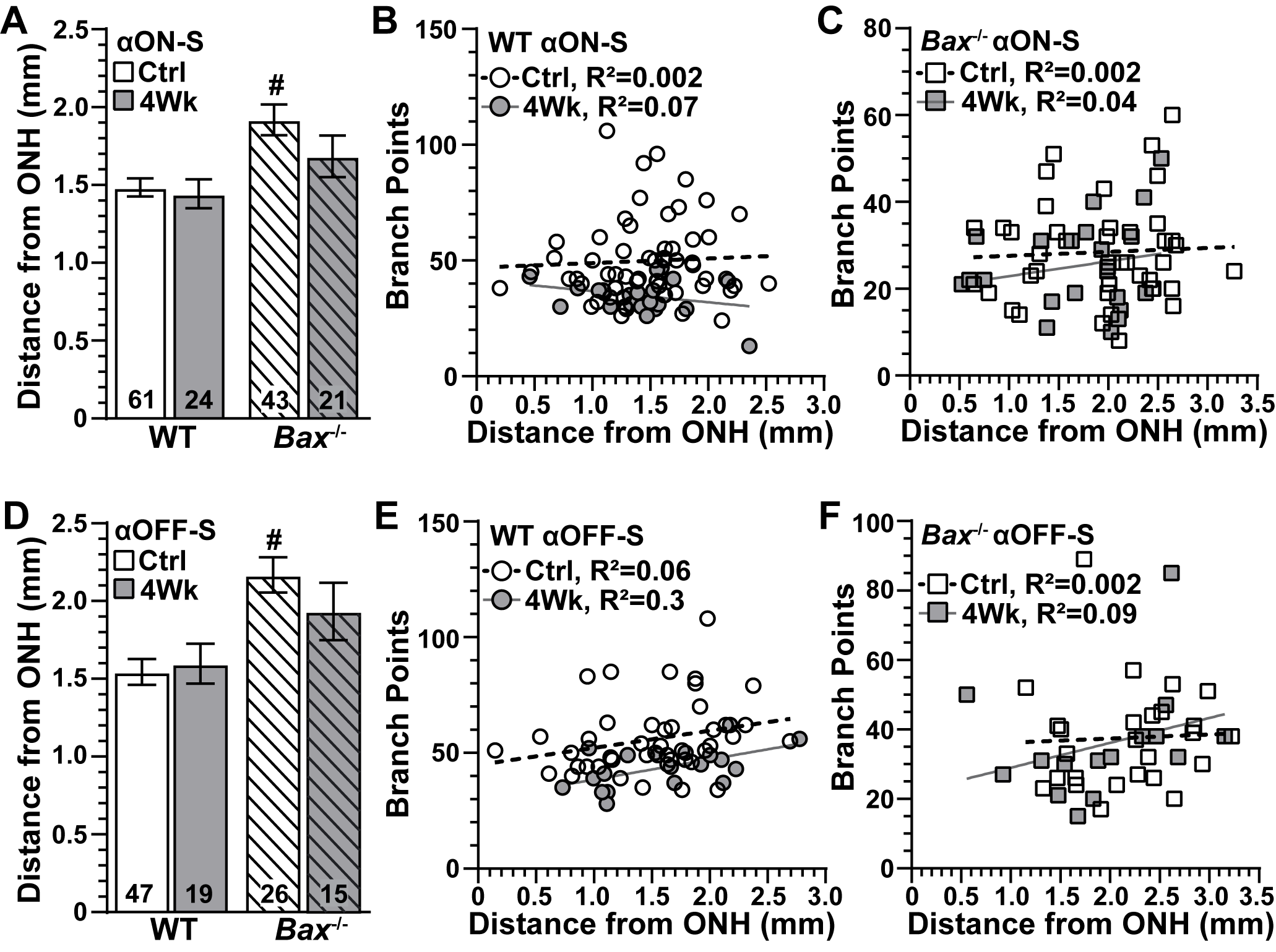

Supplement: Supplementary file 1 — αRGC Branch Points Versus Eccentricity. (A) Distance from optic nerve head (ONH) of αON-S RGCs from control and microbead-injected eyes of WT and Bax-/- mice. WT αON-S RGCs from control and microbead-injected eyes were sampled at similar retinal eccentricities (p=0.98). αON-S RGCs from Bax-/- animals were sampled at more distant locations compared to WTs (#, p=0.0005). Bax-/- αON-S RGCs from control and microbead-injected eyes were sampled at similar retinal eccentricities (p=0.36). (B-C) Relationship between branch points and distance from ONH of αON-S RGCs from WT (B) and Bax-/- (C) control and microbead-injected eyes. (D) Distance from ONH of αOFF-S RGCs from control and microbead-injected eyes of WT and Bax-/- mice. WT αOFF-S RGCs from control and microbead-injected eyes were sampled at comparable retinal eccentricities (p=0.98). αOFF-S RGCs from Bax-/- animals were sampled at more distant eccentricities compared to WT cells (#, p=0.0002). Bax-/- αOFF-S RGCs from control and microbead-injected eyes were sampled at similar distances (p=0.61). (E-F) Relationship between branch points and distance from ONH of αOFF-S RGCs from WT (E) and Bax-/- (F) control and microbead-injected eyes. Statistics: One-way ANOVA Tukey Post hoc test (A, D), Linear regression (B, C, E, F). Bar graphs indicate mean, Error bars indicate SEM. Sample number is indicated within bars. (PNG 298 kb) [file 12035_2021_2675_Fig7_ESM.png]

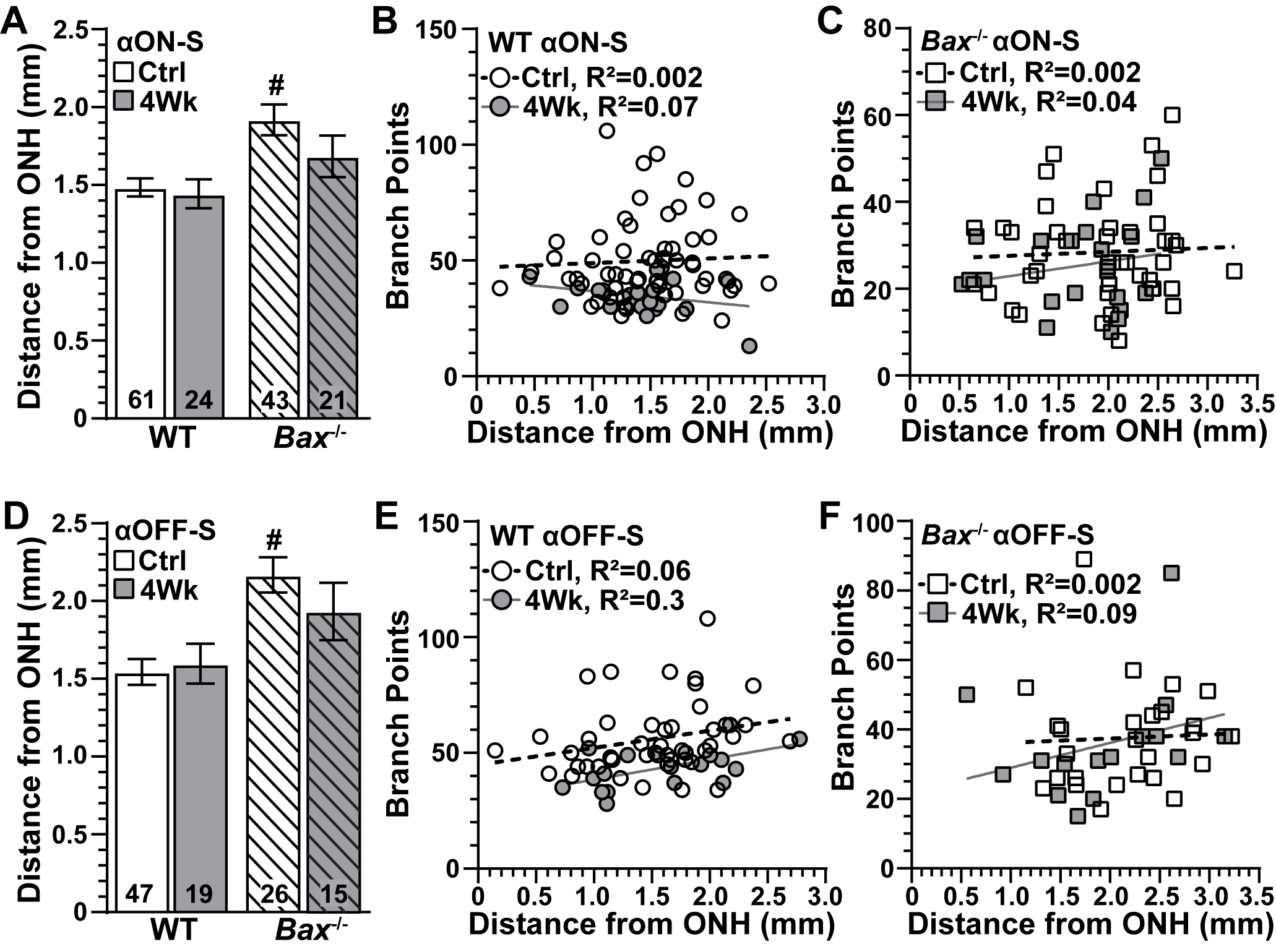

Supplement: Supplementary file 2 — High resolution image (TIF 1670 kb) [file 12035_2021_2675_MOESM1_ESM.tif]

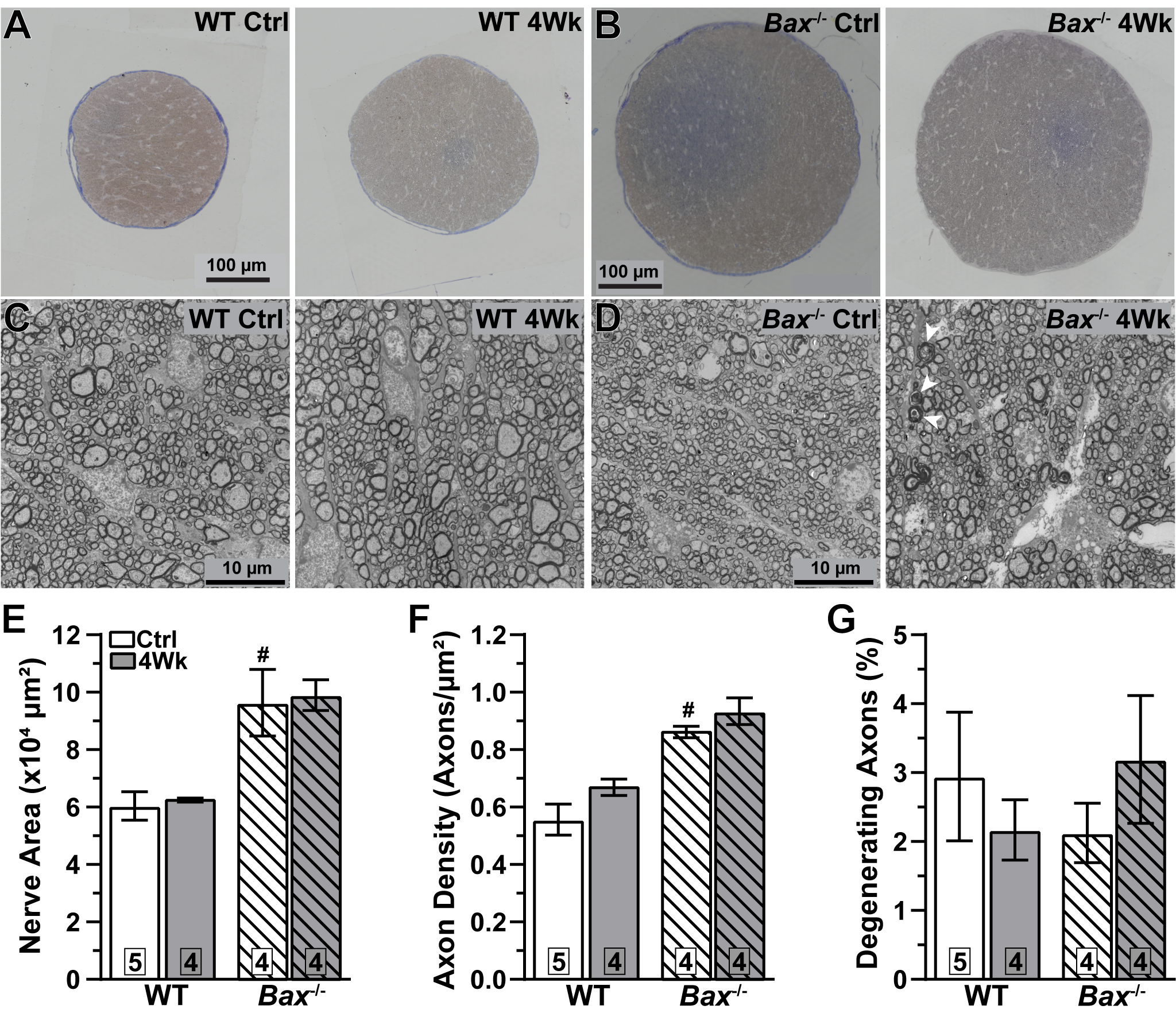

Supplement: Supplementary file 3 — IOP Elevation Does Not Affect Optic Nerve Area or RGC Axon Density. (A-B) Representative semi-thin cross-sections of optic nerves from WT (A) and Bax-/- (B) control and microbead-injected eyes. Example ultra-thin cross-section of optic nerve from WT (C) and Bax-/- (D) control and microbead-injected eyes. Example degenerating axons exhibiting multilaminar myelin sheaths indicated by arrowheads. (E) IOP elevation does not affect nerve area (p≥0.98), (F) axon density (p≥0.175), or (G) percent of degenerating axons (p≥0.767) in either WT or Bax-/- nerves. However, Bax-/- significantly increases nerve area (p=0.009) and axon density (p=0.0005). Statistics: One-way ANOVA Tukey Post hoc test (C). Bar graphs indicate mean, Error bars indicate SEM. Animal numbers: WT Ctrl n = 5, WT 4Wk n = 4, Bax-/- Ctrl n = 4, Bax-/- 4Wk n = 4. (PNG 2684 kb) [file 12035_2021_2675_Fig8_ESM.png]

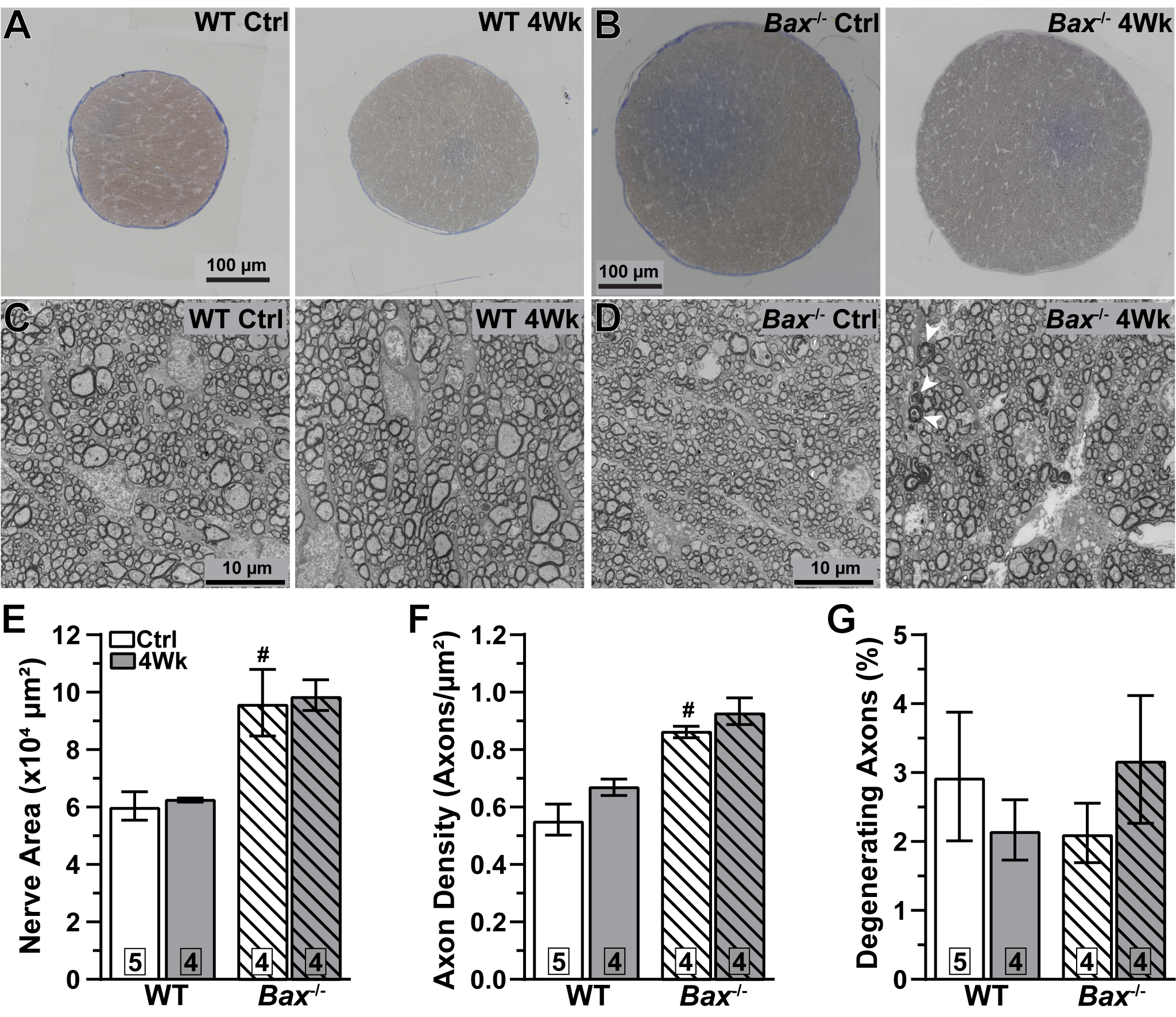

Supplement: Supplementary file 4 — High resolution image (TIF 9261 kb) [file 12035_2021_2675_MOESM2_ESM.tif]
